# A Wireless Sensor Network for Urban Traffic Characterization and Trend Monitoring

**DOI:** 10.3390/s151026143

**Published:** 2015-10-15

**Authors:** J.J. Fernández-Lozano, Miguel Martín-Guzmán, Juan Martín-Ávila, A. García-Cerezo

**Affiliations:** Departamento de Ingeniería de Sistemas y Automática, Universidad de Málaga, Calle Dr. Ortiz Ramos, s/n, Campus de Teatinos, 29071, Málaga, Spain; E-Mails: m2g@uma.es (M.M.-G.); juanmartin@uma.es (J.M.-Á.); ajgarcia@uma.es (A.G.-C.)

**Keywords:** wireless sensor network, traffic monitoring, sustainable mobility

## Abstract

Sustainable mobility requires a better management of the available infrastructure resources. To achieve this goal, it is necessary to obtain accurate data about road usage, in particular in urban areas. Although a variety of sensor alternates for urban traffic exist, they usually require extensive investments in the form of construction works for installation, processing means, *etc.* Wireless Sensor Networks (WSN) are an alternative to acquire urban traffic data, allowing for flexible, easy deployment. Together with the use of the appropriate sensors, like Bluetooth identification, and associate processing, WSN can provide the means to obtain in real time data like the origin-destination matrix, a key tool for trend monitoring which previously required weeks or months to be completed. This paper presents a system based on WSN designed to characterize urban traffic, particularly traffic trend monitoring through the calculation of the origin-destination matrix in real time by using Bluetooth identification. Additional sensors are also available integrated in different types of nodes. Experiments in real conditions have been performed, both for separate sensors (Bluetooth, ultrasound and laser), and for the whole system, showing the feasibility of this approach.

## 1. Introduction

Intelligent Transportation Systems (ITSs) are a set of solutions to improve transportation efficiency and safety [1]. There are also several studies proving how the implementation of ITS technologies enhances energy saving and reduces the emissions of vehicles [2]. Over the last few years, traffic jam problems in big cities have led to a number of research projects to address these issues. These new research projects have generated a new way of understanding Traffic Engineering.

A part of ITS systems for traffic control are based on Wireless Sensor Network (WSN) technologies [3,4]. The protocols used in these communications must be robust and fault-tolerant [5,6]. In these systems, a series of wireless sensors send the data characterizing traffic to a central unit for their monitoring and/or processing.

Traffic characterization can be made in accordance with different variables. For example, by the number of trips from one point to another, by the flow of vehicles moving on a road, or by the route followed by vehicles in their trips. For each purpose, there are different types of traffic sensors, with features which make them more or less suitable according to the traffic variable to be assessed. The type of traffic sensor to be used will also be determined by the features of the road where it will be installed on, by the possibility- or impossibility- of managing traffic interruptions and by other factors. In the following paragraphs, a classification of the sensors currently used in Traffic Engineering is made on the basis of different criteria.

Firstly, traffic sensors can be classified into two large groups according to their installation [7]:

- Intrusive sensors: they must be installed on the road, so they are expensive and usually require traffic to be shut for their installation. They give a high precision in counting vehicles. The most common intrusive sensors are inductive loops, magnetometers and pneumatic tubes.
- Non-intrusive sensors: this group includes elements which do not require installation on the road, such as radars, lasers and video cameras. In the last years, magnetic sensors which do not have to be installed under the road surface have also been developed [8,9]. They are usually more expensive than intrusive sensors.

Secondly, traffic sensors can also be classified according to the information that they get from the observed vehicles [10]. Four large groups can be made:

- Counting sensors: these sensors count the vehicles which travel through a determined control section, without distinguishing the lane or the direction. Within WSN applied to traffic control, there are initiatives based on counting sensors, such as those shown in Jeon *et al.* [11].
- Path-ID sensors: they need the installation of a wireless device in the vehicle to establish a Vehicle-to-Infrastructure (V2I) communication [12]. It is foreseeable that they will spread in the next years thanks to initiatives such as Drive C2X, EcoDrive and EcoMove of the EU [13,14,15]. Nowadays they are used in some cities so that special vehicles like buses, emergency vehicles and dangerous transportation vehicles can inform automatically of their route.
- Image sensors: they count the vehicles circulating through a certain control section and they differentiate the lane and/or the direction they are riding.
- Vehicle-ID sensors: this group covers all those sensors which are able to obtain a unique identification of the vehicle that they have detected, typically plate numbers acquired by image processing [16], or RFID readers [17]. Sensors which obtain the Media Access Control (MAC) address, such as built-in Bluetooth devices, can also be included in this group.

The diversity in the types of traffic sensors is justified by the need to obtain a certain traffic feature related to the study to be carried out and the area of interest. So, for example, if it is necessary to know the vehicle flow circulating on a certain road, it will be enough to install vehicle counting sensors. However, if the origins and destinations are required, it will be necessary to install some kind of vehicle-ID sensors [18].

The identification of vehicles’ built-in Bluetooth devices is proving to be a mature technology, able to provide interesting data in a non-intrusive way [19]. The first studies about the use of Bluetooth in traffic monitoring appeared between the years 2008 and 2010 [20,21,22,23]. Much earlier, in 2004, Bluetooth had begun to be used for Vehicle-to-Vehicle (V2V) communications [24].

The influence of the placement of Bluetooth sensors was studied in Brennan *et al.* [25] in 2010, where studies are presented on how the installation conditions with regards to the path of vehicles can modify the number of Bluetooth devices detected by the sensors. Additionally, the number of Bluetooth devices that a system is able to detect will depend as well on the total number of devices allowing to be detected. In a study carried out in Limfjord Tunnel in Denmark, it was proved that between 27% and 29% of the devices were detectable [26]. However, the studies which show data related to the percentage of vehicles with a detectable built-in Bluetooth device reach figures ranging from 1% in 2008 [22] to 7.4% in 2010 [25], and 10% in 2011 [27]. A factor which may be linked is the place where the study is carried out, as the degree of market penetration of built-in Bluetooth devices may vary from one country to another. A relation may also exist with the year of the study. For instance, Friesen *et al.* [19] report an increment of 26% in the number of unique MAC addresses over a year.

In the last years, a number of articles have been published on the important role played by Bluetooth sensors, such as Zoto *et al.* [28], who uses sensors to estimate vehicle speed, Hainen *et al.* [29], on travel times and routes, and Tsubota *et al.* [30], on traffic congestion. However, only a few references discuss using Bluetooth sensors as vehicle-ID sensors which can obtain the necessary traffic information to get origin-destination matrixes (O-D matrix), like Barceló *et al.* [20] or Hathaway and Urbanik [31]. The O-D matrix is a tool widely used in Traffic Engineering where the number of trips made between an origin and a destination is represented in matrix form. Traditionally, O-D matrixes have been obtained using counting sensors and user surveys [32]. Regarding their computation by ITS-WSN systems, some references such as Tornero *et al.* [33] make the O-D matrix calculation supposing that all the vehicles have some kind of identifier which can be recognized by the system. This assumption, even if valid given that in the short future it is planned that all vehicles have V2I communication devices, is not applicable nowadays because the proportion of wireless devices present in vehicles is not 100%, as it has been mentioned before.

This article presents a portable wireless platform, called Urban Information System (UIS) which obtains traffic and environmental information for its later monitoring and processing in a central unit, in real time. The information obtained by its sensors comprises vehicle counting, MAC addresses of vehicles’ Bluetooth devices, gases concentrations and environmental parameters like noise or dust. The processing of the vehicle count and identified MAC addresses allows the calculation of the O-D matrix in the study zone where the UIS is deployed. The main contribution of this work is the whole information system, capable of providing meaningful information for traffic managers, but particularly its capability to calculate up-to-date traffic trends thanks to the use of Bluetooth as a vehicle-ID sensor.

This article is organized as follows: after the introduction, an overview of the UIS is given, and then the architecture of each one of the sensor nodes which form the system is explained, and the communication features among the system’s components are exposed. After that, the article shows how to obtain the trends of origins and destinations relying on the proposed system and finally, it describes some of the experiments made and presents the conclusions.

## 2. Overview of UIS

The main goal of the UIS consists on characterizing the urban traffic in an area of interest. This means counting vehicles but also identifying the routes distribution, which is described by a mathematical tool named origin-destination matrix (O-D matrix). The calculation of this matrix has been implemented as an algorithm in the control system of the UIS. Besides, sensors for noise level, light intensity, pressure, temperature, humidity, airborne dust and gases concentrations are available. These sensors contribute to complement the information available to traffic managers, although they are not intended, at the current state, to enhance traffic characterization. The processed data are always accessible in real time.

The most remarkable features of the UIS are its easy deployment, low-cost, flexibility of use and energy autonomy. UIS nodes can be installed in street furniture: street lights, traffic signs…, so that construction works are not required due to its non-intrusive nature. The UIS platform is composed by smart-sensor wireless nodes whose cost is reduced in comparison with the cost of other types of counting sensor such as radar or cameras. Moreover, these types of sensors need also a high computational analysis stage to extract the useful data, increasing the required investment.

**Figure 1:**
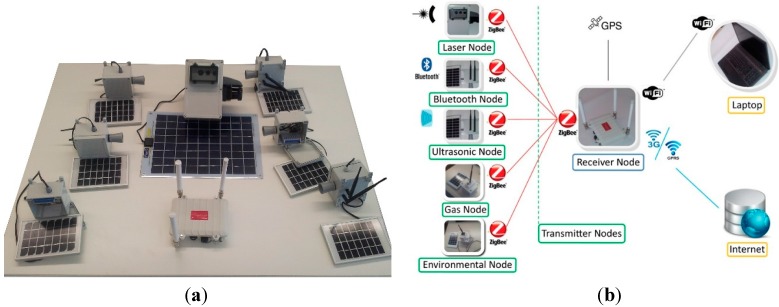
UIS platform. (**a**) General overview of some nodes; (**b**) Topology.

The modularity of the system, in the number of nodes as in the types of nodes, provides to the UIS platform the capacity to adapt its configuration to any urban area. Finally, each UIS node is fed by Li-ion batteries charging throughout photovoltaic modules in order to be totally independent from the power electricity grid. Figure 1a shows the UIS platform.

## 3. UIS Architecture

### 3.1. Topology

The basic configuration of the UIS platform comprises several transmitter nodes and at least one receiver node. The transmitter nodes collect urban environment information such as Bluetooth MACs, number of vehicles crossing the ultrasound beam, gases concentration like NO_x_, CO, CO_2_, O_2_, SH_2_, VOC, light intensity, noise or dust. Figure 1b shows the UIS topology.

**Figure 2:**
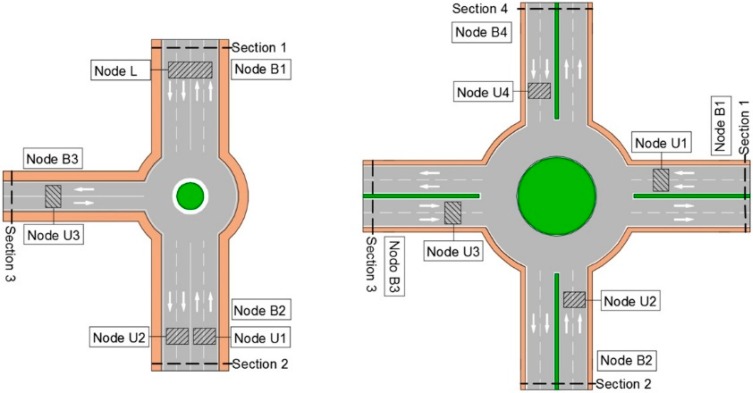
UIS deployment examples.

The flexibility of the UIS nodes and their non-invasive nature allows to adapt the UIS deployment easily to the shape of the area of interest. Figure 2 shows some examples of UIS topologies installed in different urban areas.

All the transmitter nodes share the same basic module, composed by the Waspmote V.1.2 smart platform developed by the company Libelium [34]. An XBee Pro S2 communications module, from Digi, is added to the smart platform to provide wireless capacity to communicate using protocols like ZigBee, DigiMesh, 900, *etc*. For this basic structure to be independent from the electric power grid, a solar kit has been added. The kit is made by a lithium-ion battery (either 24.42 Wh or 90 Wh) and a photovoltaic module (in two different sizes: 20 W and 1250 cm^2^; and 3 W and 168 cm^2^). Depending on the energy requirements of every node, a different configuration is used. For instance, the ultrasound node uses a 6.6 Ah battery and a 3 W panel, while the Laser node uses a 6.6 Ah battery and two 20 W panels. An evaluation of the energy autonomy is included in Section 5.1.1, as well as the configuration for every node. All these electronic components fit within an ABS plastic box with IP 67 protection so that all nodes can operate under adverse weather conditions.

In addition to the basic structure of the transmitter nodes, each node will have specific electronic components to comply with their function. The specific features of each node are described in the following sections.

### 3.2. UIS Nodes

#### 3.2.1. UIS Bluetooth Node (UIS BT Node)

**Figure 3:**
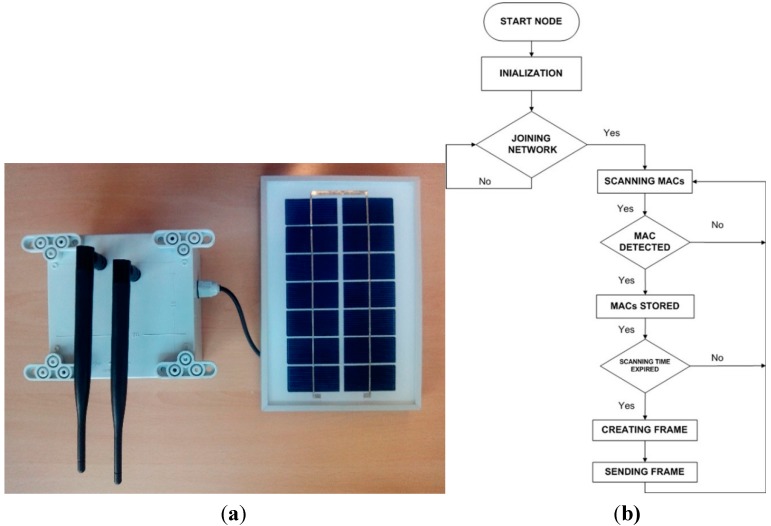
UIS BT node. (**a**) UIS BT node; (**b**) UIS BT node diagram.

The Bluetooth node can be described as a non-intrusive, vehicle-ID smart sensor. The sensor used for detecting Bluetooth devices is a BLUEGIGA WT12 Bluetooth module added to the Waspmote V1.2 platform mentioned above. It has a radio expansion connected to socket 0. The node is presented in Figure 3a.

The detection of Bluetooth devices in vehicles is an important aspect of this work. For this reason we proceed to explain the process by which our sensory system nodes detect the MAC address of the devices found.

The Bluetooth sensor module is continually seeking to join a possible Bluetooth network, starting a cycle every five seconds to detect all potential devices to connect with. When a Bluetooth device of the vehicles within the range of the sensor receives a connection request, it automatically responds by providing information on the Media Access Control (MAC), the manufacturer and Class of Device (CoD). The UIS uses this information as follows:

- MAC: It is unique for each Bluetooth device manufactured in the world. It identifies a device from any other unequivocally. Since users can decide whether their systems are visible or not, there are no privacy issues. However, if necessary, a part of the MAC address can be considered, instead of the whole identification.
- CoD: It describes the type of device (*i.e.*, hands-free, smartphone, laptop, *etc.*). It helps to decide whether a certain MAC should be considered as belonging to a vehicle or not.

Figure 3b shows how the UIS BT node works. The node, once initialized, tries to join a UIS network. If the parameters’ setup corresponds to those of the UIS network (PANID, channel, Linkkey, *etc.*), then the node joins it. If not, it will keep on looking for a suitable network. Once the node is properly joined, the UIS BT node starts to look for Bluetooth devices to detect their MACs. After five seconds, the detected MACs will be stored in the internal memory of the Waspmote V1.2 platform. A frame will be created, implying processing, packaging and encryption, and then it will be sent to the receiver node as a frame of the ZigBee protocol.

#### 3.2.2. UIS Ultrasound Node (UIS ULT Node)

**Figure 4:**
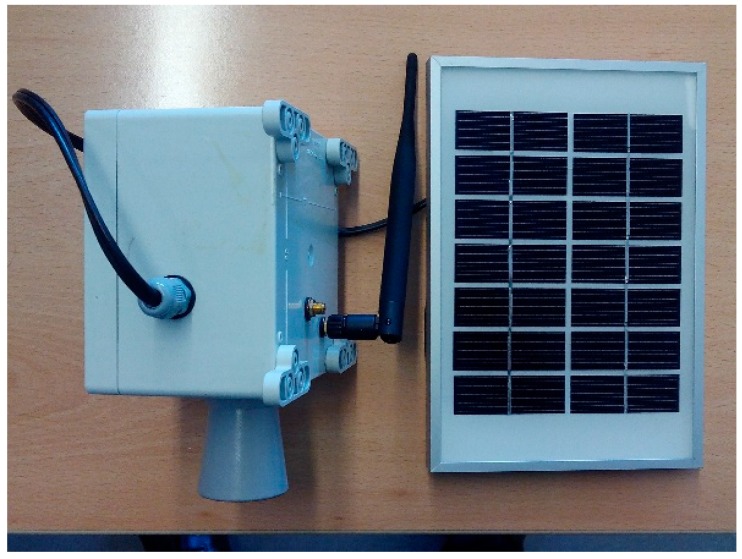
UIS ULT sensor.

The ultrasound node acts as a non-intrusive, vehicle counting, smart sensor. It is built around the basic module shared by all the transmitter nodes and a *Smartcities* board developed by Libelium and which incorporates the electronics needed to interface with the Maxbotix XL-MaxSonar-WR1 ultrasound sensor (Figure 4).

**Figure 5:**
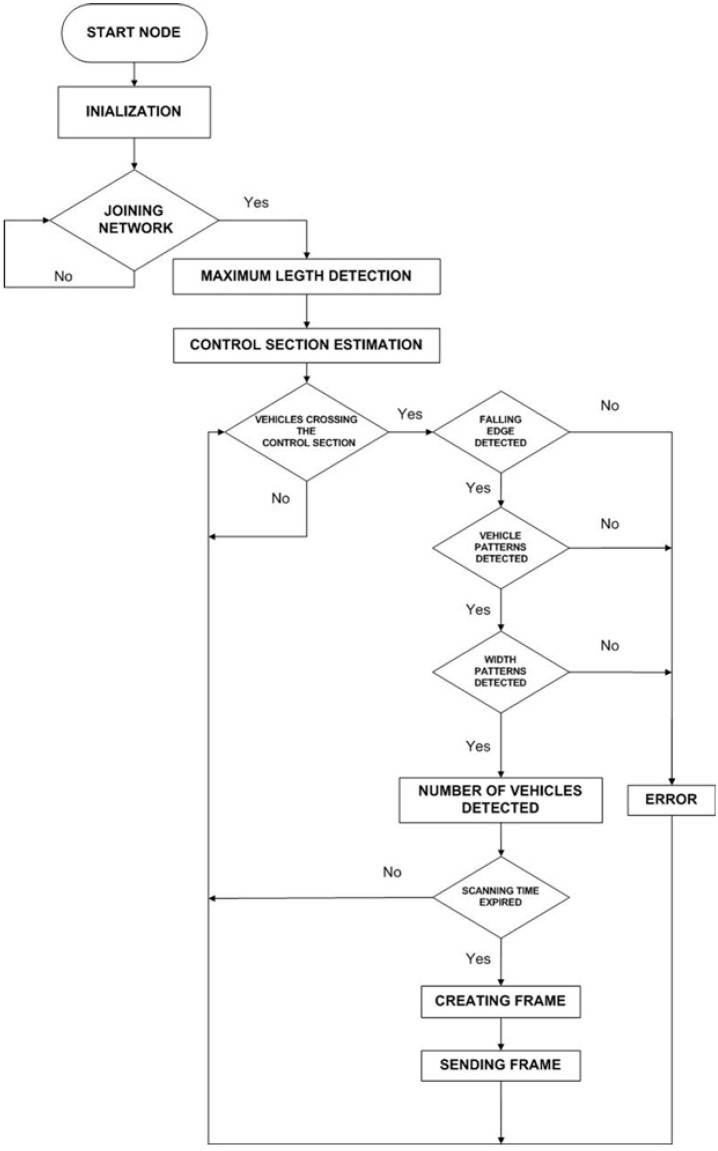
UIS ULT node diagram.

Basically, this node detects the number of vehicles passing through its ultrasound beam during a scanning time of five seconds. After that time, it sends the detected number of vehicles to the receiver node through a ZigBee frame. Figure 5 shows a diagram of the process. After an initialization, the node tries to join the UIS network. On succeeding, it configures the parameters used to detect vehicles: the maximum length detection (obtained by in-site calibration), and the control section length estimation (depending on the deployment of the system). When a vehicle crosses the control section, a falling edge is detected. The rising edge marks the width of the pulse, which is compared to a heuristic parameter ΔT which depends on the speed on the road under study. Based on the width of the pulse compared to ΔT, vehicles are counted. Finally, the number of vehicles is reported to the receiver node as a string. This requires a very short time compared to the sampling period of the signal from the ultrasound sensor (0.2 s), so the possibility of missing a vehicle is minimized.

#### 3.2.3. UIS Laser Node

The laser node acts as non-intrusive and image smart sensor. This node has the main function of counting the vehicles passing through a certain roadway and, additionally, is capable of identifying on which lane of the studied roadway the counted vehicle is riding. This last feature provides wide advantages with regards to the traditional vehicle counting systems, such as the pneumatic tubes. Besides, since vehicle identification is made by image processing techniques, it is possible to obtain geometric characteristics of the counted vehicle, such as its height and width. With those two characteristics, vehicles can be classified into three groups: passenger cars, heavy vehicles and two-wheeled vehicles.

**Figure 6:**
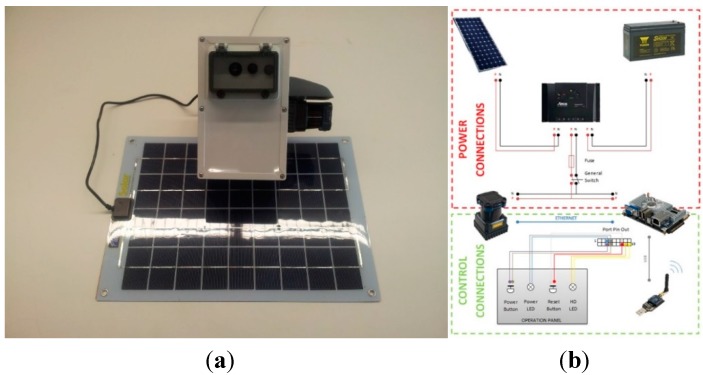
UIS Laser node. (**a**) UIS Laser node; (**b**) UIS Laser node hardware.

The installation scheme of the laser node is represented in Figure 6. The main elements are the laser sensor, the processing unit and the communication modem. The main features of each element are described below:

- Laser sensor: Hokuyo UTM-30 LX-EW, class 1 2D scanner laser range finder.
- Processing unit: a Pico-ITX board.
- Communication modem: a ZigBee module is used to interface with the rest of the elements of the UIS.

Figure 6a shows the laser node in its real implementation, with the photovoltaic module connected. Figure 6b shows a general diagram of the different hardware elements.

**Figure 7:**
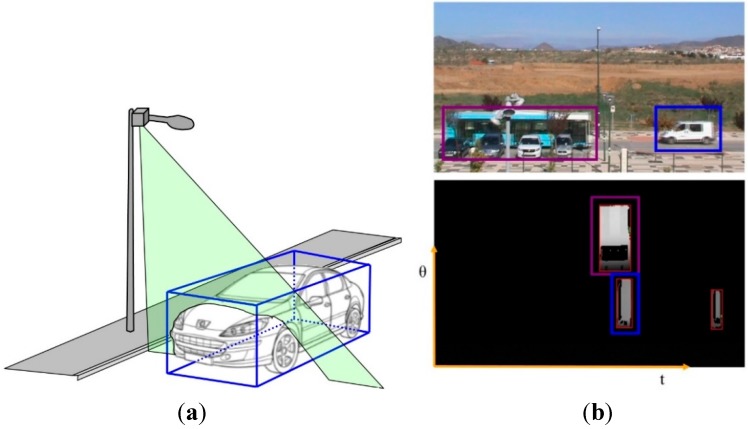
UIS Laser node. (**a**) UIS Laser node beam; (**b**) Video image (top) corresponding to a laser node image (bottom).

The laser node is placed in elements of the street furniture such as lighting poles or any other element with a height between 7 m and 9 m. The node is placed so that the laser beam is oriented perpendicularly to the roadway and the direction of the vehicles, see Figure 7a. This way, the successive scans of the laser sensor result in a series of points which can be sorted in an equivalent image, see Figure 7b. The equivalent images represent time in one axis, the angle in relation to the laser sensor origin in another axis and, finally, each pixel represents the height with regards to a reference in the color intensity. With those details, vehicles are detected using image processing techniques.

#### 3.2.4. Additional Sensors

Besides the sensors described in the different nodes above, some other sensors are available. Their goal is not to contribute to traffic characterization, but to obtain additional information usually relevant to the same traffic managers interested in traffic trend monitoring. These nodes are the UIS Gas node and the UIS Environmental Pollution (EP) node.

**Figure 8:**
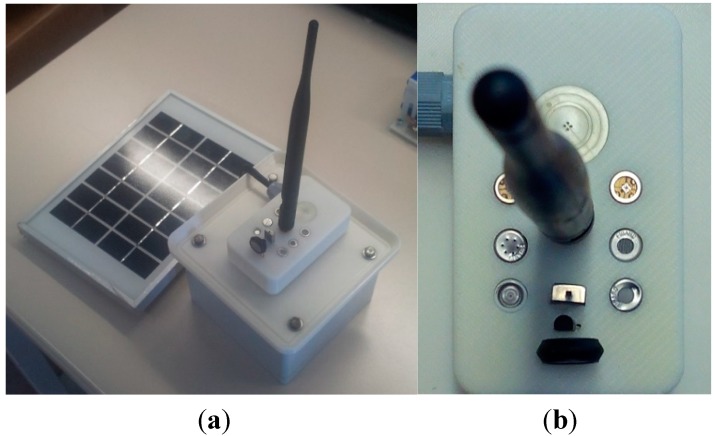
UIS Gas node. (**a**) UIS Gas node; (**b**) Gases module.

The Gas node includes sensors for concentration of gases such as O_2_, CO_2_, CO, NH_3_, VOC…, as well as pressure, temperature and humidity, see Figure 8a. To take the data from the gas sensors, the module shown in Figure 8b has been developed. The used sensors are:

- Humidity sensor: J808H5V5 Humidity transmitter, from JIN ZON ENTREPRISE CO.
- Atmospheric Pressure sensor: MPX4115A, from Motorola.
- Temperature sensor: MCP9700/9701, from Microchip.
- O_2_ sensor: SK-25, from Fígaro.
- O_3_ sensor: MICS-2610, from E2V.
- CO_2_ sensor: TGS 4161, from Fígaro.
- CO sensor: TGS 2442, from Fígaro.
- NH_3_ sensor: TGS 2444, from Fígaro.
- VOC sensor: TGS 2600, from Fígaro.

The EP node includes sensors for light intensity, noise level and airborne dust:

- Dust sensor: GP2Y1010AU0F, from Sharp.
- Light intensity sensor: GL5528 photo resistor.
- Noise sensor: WM-61a, from Panasonic.

**Figure 9:**
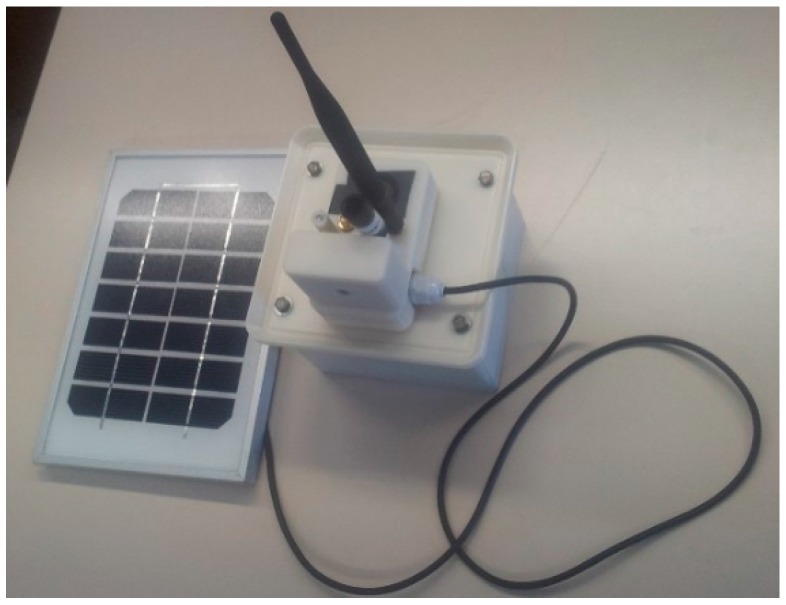
UIS EP node.

To interface them with the Waspmote V1.2 basic module, a Smartcities board, from Libelium, has been included. Figure 9 shows the real EP node, with its photovoltaic module connected.

#### 3.2.5. UIS Receiver Node

The Receiver node is in charge of creating the UIS network for it to be joined by the rest of the UIS nodes. For that purpose, it acts as a ZigBee coordinator defining the PAN ID (Personal Area Network identifier) of the network, the transmission channel according to its energy saving algorithm, and the security policy. Only those sensor nodes meeting the previously mentioned requirements will be able to join the UIS network.

Apart from creating the network and allowing other nodes to join in, the Receiver node has the capability to store the information, sent as frames, of the transmitter nodes which have joined the network. These frames will be processed by the Receiver node extracting from them the data needed from each sensor, and storing its value in the sensors’ data table of the internal SQL database of the node itself.

Additionally, this node allows synchronization with a database in an external server through 3G communications, and allows as well to send to that database the table with the sensors’ data, already processed for them to be used as required, for example as inputs for a SCADA system.

**Figure 10:**
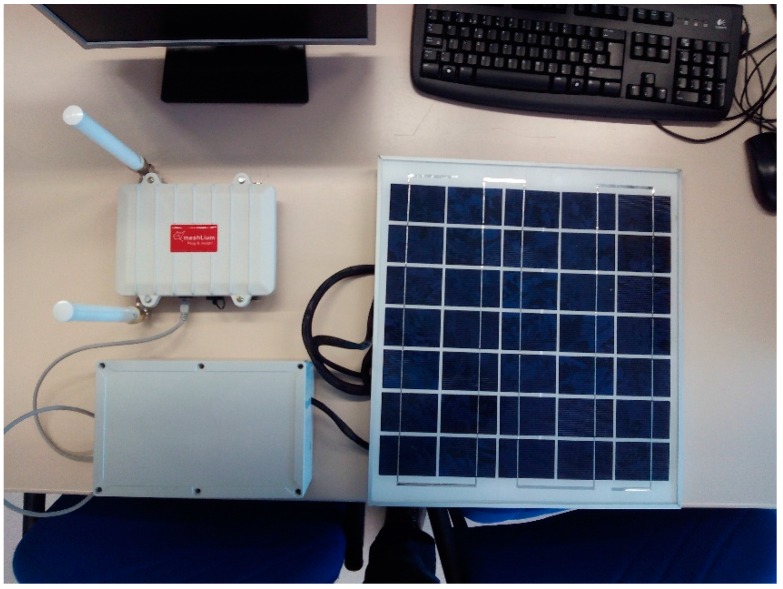
UIS Receiver node.

The UIS Receiver node is shown in Figure 10, and has the following components:

- Meshlium modem, from Libelium.
- Photovoltaic module, specific for Meshlium modem, from Libelium.
- 12 V battery.

**Figure 11:**
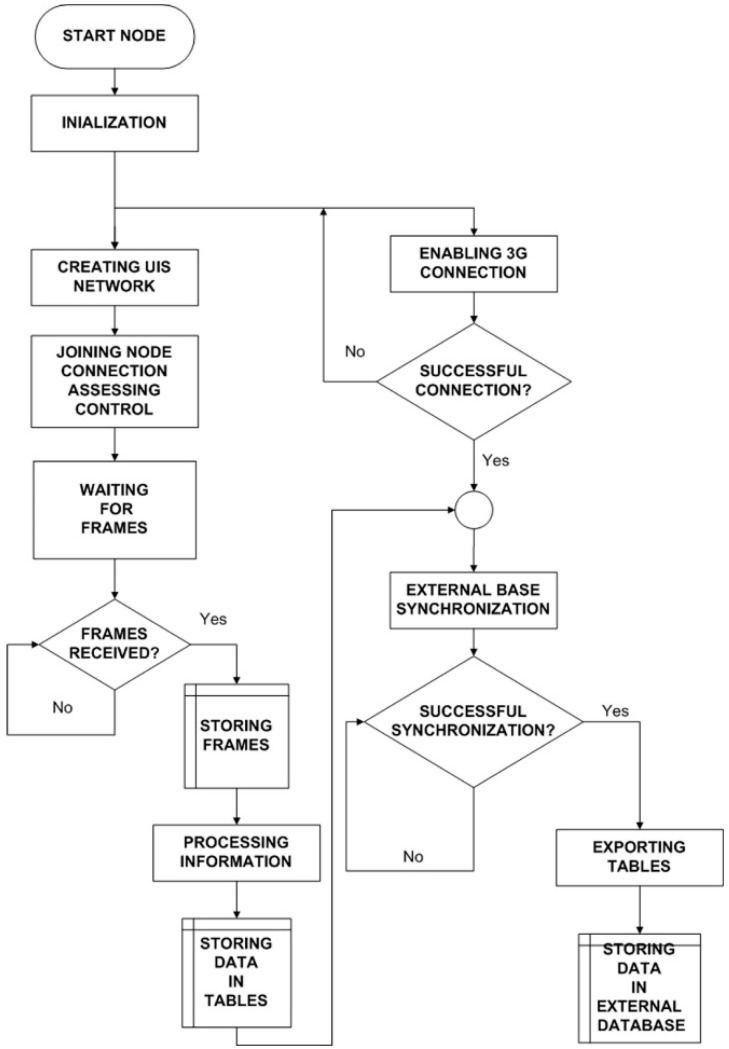
UIS Receiver node diagram.

The diagram of the node can be seen in Figure 11. Besides creating and coordinating the UIS network, the Receiver nodes extracts the data from the received frames, and stores them in its internal database. This database is synchronized with an external one in a server, connected via 3G.

### 3.3. Communication

The ZigBee protocol has been selected because of its technical features [35]. It allows to send data packages of up to 65 Mb at high speed, and with the possibility to defragment the main package in several sub-packages if they exceed a size of 65 Mb. Also, the transfer of information is made with little energy consumption and, therefore, it can have a higher autonomy.

However, this standard has a low transfer rate: from 20 kb/s to 250 kb/s. The range for point-to-point communications goes from 600 m to 7 km, although it depends on the obstacles between the emitter and the receiver. Depending on to the network topology, the number of nodes may range from 256 to 64770. Also, the ZigBee standard includes features to increase robustness. For example, if a node falls, the other nodes which depend on it will search for other neighbor nodes to join to and to continue being member of the network.

**Figure 12:**
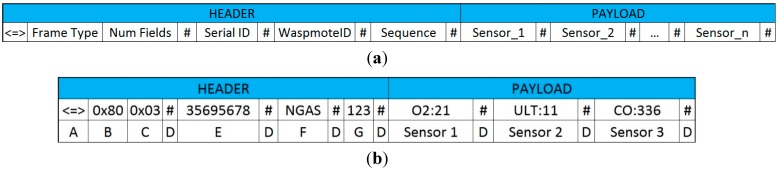
ASCII frame. (**a**) Frame structure; (**b**) Example with sensor data.

Within the different frame types for the ZigBee protocol, an ASCII frame structure has been selected for this application. The frame structure can be seen in Figure 12, where:

(A) Start delimiter (3 bytes): first field of the frame made by three characters “<=>”.

(B) Frame-type indicator byte (1 byte): this field defines if the frame is binary or ASCII form.

(C) Number of sensor fields (1 byte): it specifies the number of sensor fields to be included by the sending frame.

(D) Separator (1 byte): the start of each field is marked by “#”.

(E) Waspmote identifying number (10 Bytes): this field is made by a 10-bit-long numeric string which identifies with a single number each V1.2 Waspmote platform. It is integrated by default in the V1.2 Waspmote chip and it cannot be modified.

(F) WaspmoteID (0–16 bytes): string of characters which identify the V1.2 Waspmote platform. This field can be modified by the programmer.

(G) Frame sequence (1–3 bytes): it indicates the sequence number of any frame sent. It is made by 8 bits and numbered from 0 to 255. When the value 255 is reached, the counting resets to 0.

Sensor (i): specific field of each sensor with the information that it provides in accordance with its programming. Its length can be variable.

The different frames used in the UIS have the same header and they differ in the data packages that they carry. For example, Table 1 shows a UIS BT node frame. The Waspmote V1.2 value appears first, in the field WaspmoteID. The second field is the ID, defined as N1BT (meaning node 1, Bluetooth type). Frame Type shows the number of the frame in the series. Then, the payload presents the type of sensor (where MAC means a MAC identifier sensor, *i.e.*, a Bluetooth node), the data (the detected MAC address), the RSSI and the device type.

The creation of the UIS Network is initiated by the Receiver node assuming the role of coordinator node of the ZigBee protocol. It selects the channel value for the transmission, the energy policy, the PANID, the extended PANID and the security policies.

Later, all those nodes whose configuration parameters match those defined by the coordinator node and which request to join the network, will be able to do so after being accepted by the coordinator node.

Regarding security, frame authentication, encryption and validation are included. The encryption phase is made by hardware, using the AES-128 algorithm (AES, Advance Encryption Standard).

**Table 1:** UIS BT node frame.

Waspmote ID	ID	Frame Type	Payload			
366334232	N1BT	26	MAC	50:2d:1d:fb:78:2f	−80	P

### 3.4. SCADA

**Figure 13:**
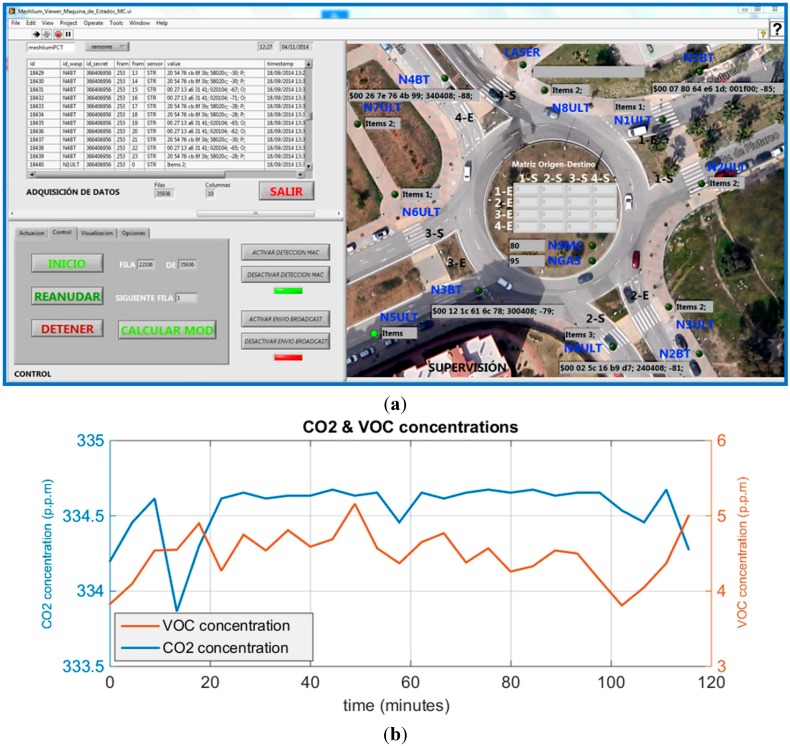
(**a**) SCADA; (**b**) Historical graph for gases.

The information gathered from the area of interest and stored as tables in the internals database of the UIS Receiver node is sent as well to a database in an external server, and it is synchronized with the UIS Receiver node via 3G communication. This database is the input for a SCADA system developed for this application and which allows to use the stored information to carry out supervision tasks. As it can be seen in Figure 13a, considering the deployment of the UIS in a certain area, it is possible to know the frames in real-time, and from them, it is possible to show the information obtained by each sensor. For example, Figure 13b shows a historical graph of gases and environmental pollution parameters detected in a real test of the system.

## 4. Travel Trends: Origin-Destination Matrix

One of the main contributions of the proposed UIS is the real-time calculation of the O-D matrix of the observed area. The O-D matrix characterizes the trips made in a certain region, where each element *ODM_ij_* represents the trips with origin in *i* and destination in *j*. In short, it can be described as:

$$ODM=\left[\begin{array}{ccc} \beta_{11}\cdot E_{1} & \cdots & \beta_{1n}\cdot E_{1}\\ \vdots & \ddots & \vdots \\ \beta_{n1}\cdot E_{n} & \cdots & \beta_{nn}\cdot E_{n} \end{array}\right]$$

where *E_i_* represents the total of vehicles with origin *i*, and *ß_ij_* represents the proportion of vehicles with origin *i* and destination *j*. This proportions can be written as a matrix *B*:

$$B=\left[\begin{array}{ccc} \beta_{11} & \cdots & \beta_{1n}\\ \vdots & \ddots & \vdots \\ \beta_{n1} & \cdots & \beta_{nn} \end{array}\right]$$

Determining the values of these elements for a given time has been out of the reach of the available technology, especially with regards to matrix *B*. Even if it is possible to know with a reasonable accuracy the number of vehicles departing from or arriving to a certain place (for example, using pneumatic tubes), knowing the proportion of vehicles travelling between two points *i* and *j* has had to be made by estimations, usually through the data obtained in roadside interviews. The time required to get those data and the difficulty to do so reliably have driven a certain amount of works proposing different ways of estimating the matrix *B* [36].

The proposed system allows to find the proportion of vehicles with origin in *i* and destination *j* through the identification of the Bluetooth devices’ MAC. By completing with the data obtained by the ultrasound sensors, which allow to acquire *E*, it is possible to calculate in real-time an O-D matrix.

For that purpose, the Bluetooth nodes collect the MACs within their range and send them periodically to the coordinator node within a frame. The coordinator node synchronizes all that information with the SQL database of the monitoring and processing central unit via 3G connection.

First of all, the existing Bluetooth frames are filtered. The nodes send frames with all the MAC addresses found, and a part of them may belong to close devices and not to a vehicle. For example, signals from a laptop close to a node, wireless speakers of a shop, *etc.* Also, MAC addresses which have been detected only once are also identified and eliminated from the process of obtaining the O-D matrix, because with them it is not possible to identify an origin and a destination.

Each Bluetooth node is associated to a control section, what involves a determined origin (and/or destination), so that when a node sends a frame its ID is known and, therefore, its location. It must be assured that a vehicle is detected by only a Bluetooth node at a time, so that it is possible to establish its origin and its destination. This premise is remarkably important for the first and last detection of any vehicle, and has to be taken into account when deciding the deployment of the nodes.

The first valid MAC of the database is a candidate to be an origin of a trip. Later on, the other frames are studied until the last appearance of that MAC is discovered, and therefore, the destination of that trip is known. The processing to do so relies on two main criteria to determine when a valid MAC reaches its destination (*i.e.*, leaves the control area):

- That a time longer than the characteristic time between the origin and destination has passed (“inc_max”). The value of this parameter will depend on the average time that it takes for a vehicle to cover any possible itinerary, plus a security time.
- That a certain number of frames has been analyzed (“frames_max”). This parameter is introduced to limit the processing, and depends on the number of frames received per time unit. This is a function of the number of installed sensors and the vehicle flow in the study area.

The tuning of these parameters is made experimentally. They depend on different characteristics of the control area, such as:

- Average vehicle speed.
- Distance between the Bluetooth nodes.
- Number of nodes installed in the control area.

**Figure 14:**
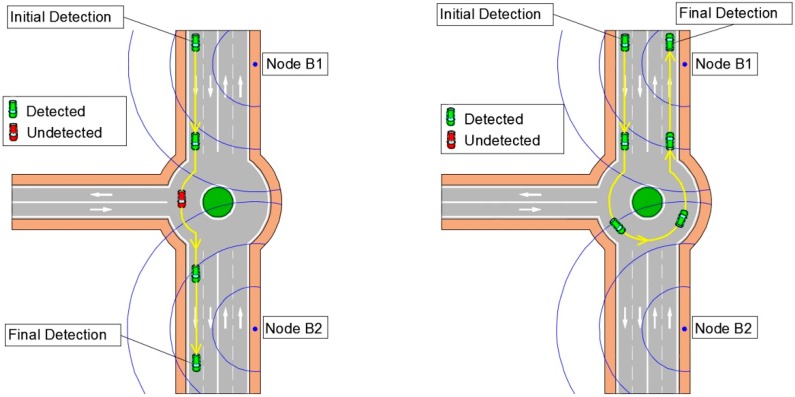
Vehicle detection using Bluetooth MACs.

Figure 14 shows a simplified scheme of the process to identify origins and destinations in a control area.

In the figure, a vehicle with a detectable MAC is identified when entering the control area of the system in the surroundings of the B1 node. According to its speed, it will be detected at other times by the same or by other nodes until it leaves the control area. The algorithm tries to find the first and last frame with that MAC, and to obtain the entry node and the exit node, updating the corresponding element in the matrix of origin-destination trends *B.* Martín-Guzmán *et al.* [18] explains in detail how to obtain the O-D matrix, besides some preliminary work.

## 5. Experimental Validation

Several tests have been carried out to verify the performance of the UIS. On the one hand, we have carried out independent tests of the different nodes. On the other hand, we have completed different integration tests, including experiments in real roadways.

### 5.1. Tests on the UIS BT Node

#### 5.1.1. Indoor Tests

This test was carried out with a double purpose: to prove the capability of the node to detect Bluetooth devices, and to check if it was possible to track the MAC of a certain Bluetooth device.

To perform the tests, three Bluetooth nodes were deployed in the surroundings of the Library of the Escuela Técnica Superior de Ingeniería Industrial, in the Universidad de Málaga, covering the trajectory of a corridor: In this case, instead of built-in Bluetooth aboard vehicles, the goal was detecting Bluetooth from smartphones. Both tests were a success, because many Bluetooth devices were detected simultaneously and it was also possible to follow a specific device by tracking its MAC in the different Bluetooth nodes deployed.

Besides these tests, the energy autonomy was also evaluated. The energy consumption of all nodes was measured around a 24 h cycle. Table 2 shows these data besides the available energy in the batteries (considering 80% of their full capacity) and the contribution of the solar panels (considering an average solar irradiance of 5.3 kWh/m^2^ d for the area of the experiments [37]). It can be seen that the contribution of the solar panels is sufficient to provide the required energy for one day. Besides, the battery can deliver energy enough to keep the system working during the night and, also in the event of low solar contribution.

**Table 2:** Energy consumption of the UIS nodes.

	Configuration	Measured Energy Consumption (Wh/d)	Battery Contribution (80%, Wh)	Solar Panel Contribution Per Day (Wh)
ULT Node	24.42 Wh battery, 3 W panel	5.68	19.54	15.89
BT Node	24.42 Wh battery, 3 W panel	8.35	19.54	15.89
Gas Node	24.42 Wh battery, 3 W panel	12.70	19.54	15.89
EP Node	24.42 Wh battery, 3 W panel	5.68	19.54	15.89
Laser Node	24.42 Wh battery, 2 × 20 W panels	172.80	72.00	264.89
Receiver Node	24.42 Wh battery, 20 W panel	124.20	72.00	132.45

#### 5.1.2. Outdoor Tests

A second series of tests had as its goal to check the proper working of the system outdoor, in more real environments, as well as to check the percentage of vehicles with installed Bluetooth devices.

**Figure 15:**
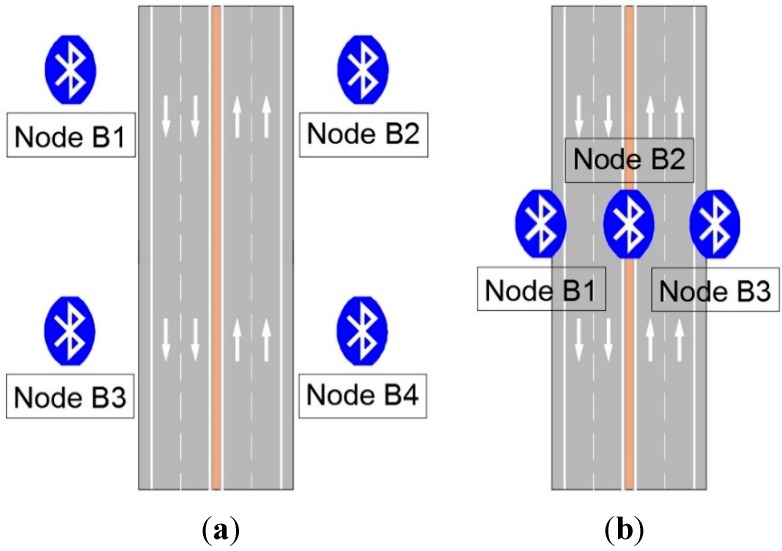
Different deployments for outdoor tests. (**a**) A-357 deployment test; (**b**) Valle-Inclán Av. deployment test.

The chosen area was a section of the Highway A-357 in Malaga (Spain), with a speed limit of 100 km/h. Four Bluetooth nodes were deployed, two on each roadway direction, and a receiver node to control the data flow of the UIS network, as in Figure 15a.

The total number of vehicles passing through the control section was obtained by counting on a video recording of all experiment time. The result obtained by the UIS was a detection of 12% of vehicles (see Table 3). Those data match the figures published in other recent works, such as Brennan *et al.* [25].

**Figure 16:**
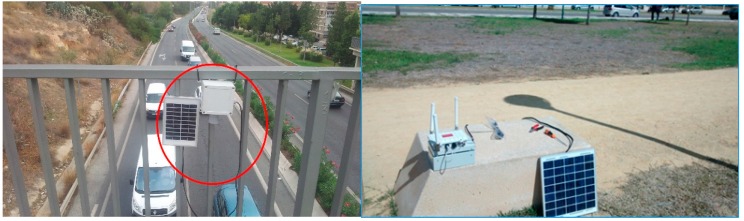
Different installations for UIS nodes. (**a**) UIS ULT node installation; (**b**) UIS Receiver node with solar kit.

These tests were repeated in other areas with traffic with different characteristics. For example, in the Valle-Inclán Avenue, where speeds range between 80 and 40 km/h. A different deployment was chosen, using a bridge over the roadway to deploy the nodes as in Figure 15b. The real number of vehicles was also verified by video recording. This time, the percentage of detected vehicles was 9.88% (see Table 3). Detection rates in this Table take into account received frames, so they include errors in detection and transmission. Despite the differences in the speeds of vehicles in the studied roads, the rates of identified vehicles are similar. Figure 16 shows images of installation for different UIS nodes.

**Table 3:** Detected vehicles in Bluetooth tests.

	Vehicles Counted	Number of Received Frames	Unique MAC Address Detected	Percentage of Detected Vehicles
Roadway A-357	3580	1155	477	12.48%
Valle Inclán Av.	5868	1535	555	9.88%
**Total**	9448	2690	1032	10.92%

**Figure 17:**
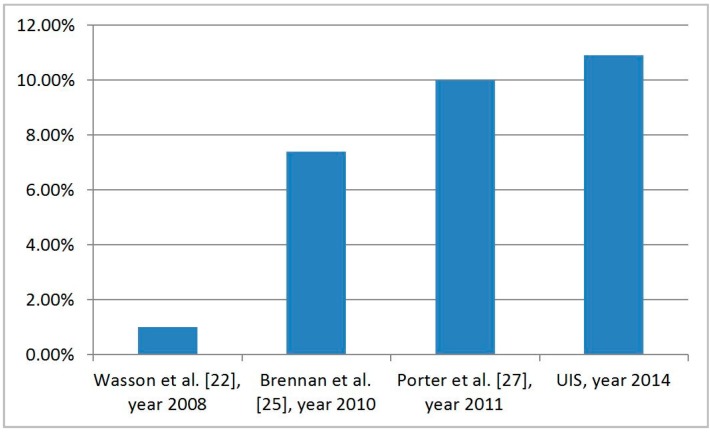
Comparison of the proportion of vehicles detected using Bluetooth according to different authors.

It is worth comparing the obtained result to those of the literature. It can be seen in Figure 17 that the number of detected vehicles has been increasing during the last years, and the proportion of detected vehicles is around 10%. This means that Bluetooth identification can obtain data from 1 out of every 10 vehicles, in a proportion that no direct interviews can match.

### 5.2. Tests Carried Out with the UIS ULT Node

**Figure 18:**
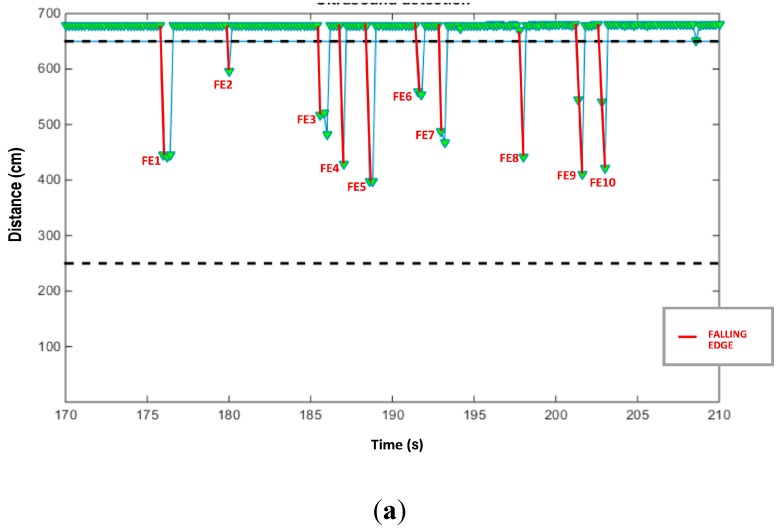

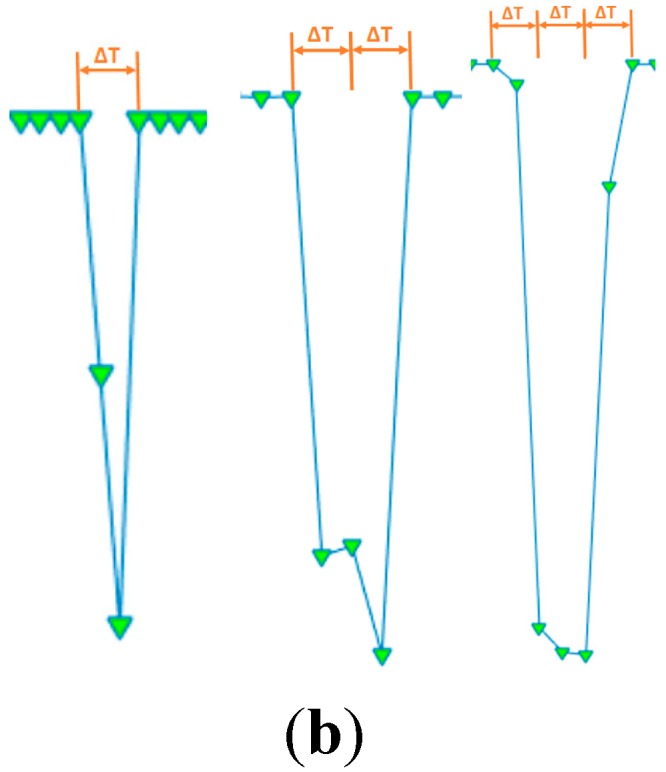
UIS ULT node test. (**a**) Ultrasound detection; (**b**) Detection of different kinds of vehicles through pulse width.

Even if the main sensor of the UIS is the Bluetooth, especially with regards to vehicle identification, it is necessary to have a sensor counting the total number of vehicles. In the case of the UIS, a node with ultrasound sensor was implemented (UIS ULT node). This node is installed in such a way that its control section is perpendicular to the roadway. The node only sends frames if any vehicle is detected, in order to minimize the consumed energy and the network traffic. This detection is based on the detection distance and the time this detection is active, and rising and falling edges are also taken into account to eliminate errors (such as a vehicle stopped next to the sensor’s control section due to jams). When a vehicle crosses the ultrasound beam, a falling edge is detected in the signal, marking a candidate, as shown in Figure 18a. The time until a rising edge is detected is compared with a parameter ΔT, depending on the average speed in the particular road, see Figure 18b. This provides with the data to decide whether a vehicle was detected (beyond a minimum width of the pulse).

Several experiments were carried out in real streets. Figure 18a shows data from one of them. The sensor was installed during two hours, and counted 2324 vehicles. A video recording of the whole period showed a real figure of 2368 vehicles, which means an accuracy of 98%.

### 5.3. Tests Carried Out with the UIS Laser Node

**Figure 19:**
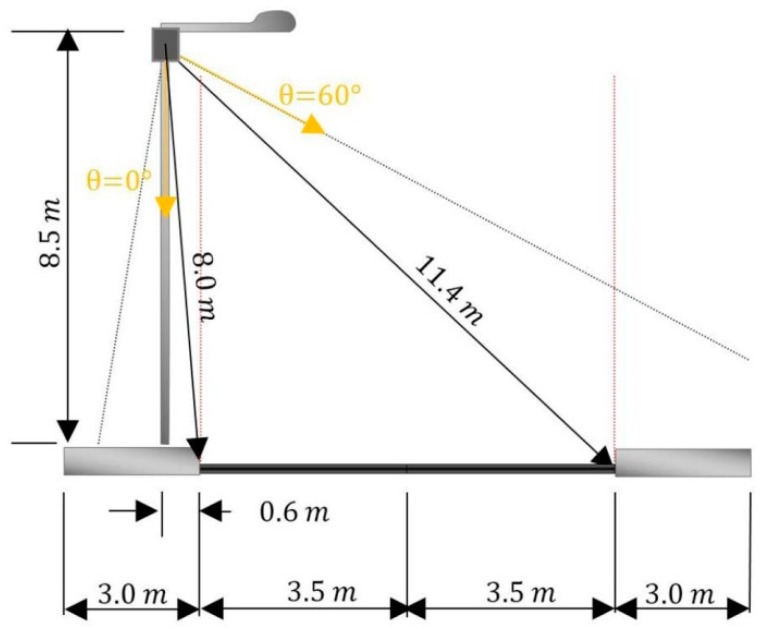
UIS Laser node installation.

This node has also been validated on real roadways. The final test took place in a street with two lanes, one for each direction, and to walk sides, one at each side of the roadway. The laser node was placed on a lighting pole of the roadway, oriented towards the floor, as it can be seen in Figure 19. The test took place for 1 h 30 min. During that time, equivalent images of 15 s were obtained for its later analysis at the laboratory.

During this experiment, a total of 238 vehicles were counted, and 203 vehicles were detected correctly, an accuracy of around 85%. Errors were mainly caused by very dark cars circulating, mostly, by the lane most distant from the sensor. The intensity of the infrared radiation which, under these circumstances, reaches the sensor is not enough to be measured considering the power and sensibility of the chosen sensor. Regarding the determination of the circulating lane, efficiency was 100% of the detected vehicles.

### 5.4. UIS Tests

**Figure 20:**
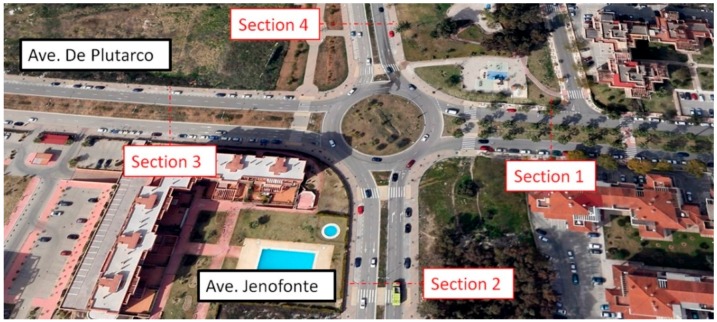
Experimental set up.

**Figure 21:**
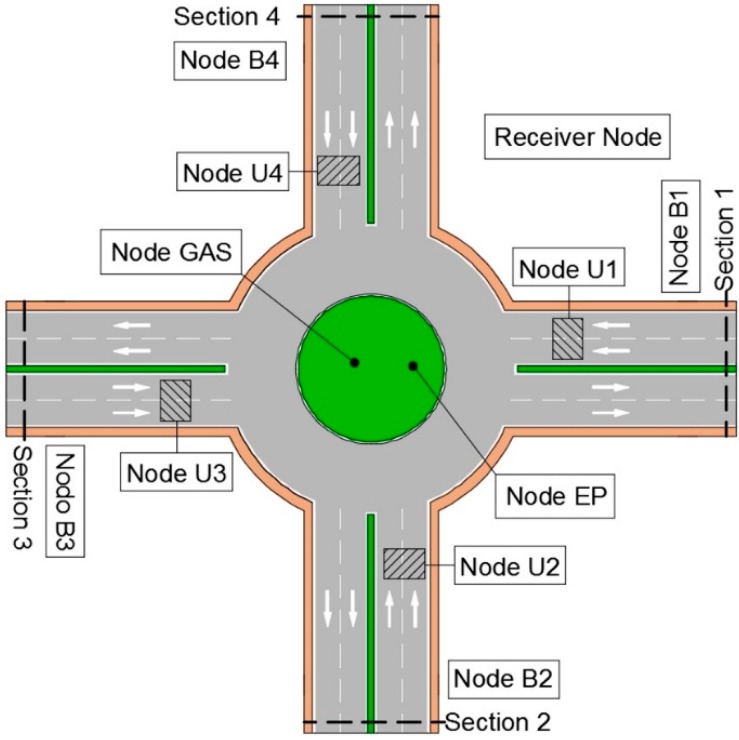
Experimental distribution of nodes.

**Figure 22:**
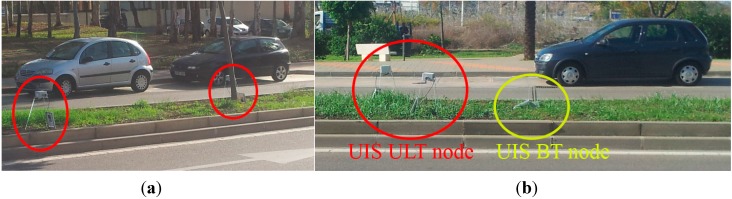
Some UIS nodes in a real experiment. (**a**) UIS nodes in roundabout I; (**b**) UIS nodes in roundabout II.

As well as the different integration tests, made at the laboratory, experiments in real roadways have been carried out. For one of them, different types of UIS nodes were deployed, as seen in Figure 20, in the surroundings of a roundabout. This zone for the experiments was selected because it provides with different entries and exits within a short distance, but the system could be deployed in distant locations with no significant differences. The area of study was divided into four control sections. In each section, two UIS ULT nodes where deployed to count the number of total vehicles in each lane, and an UIS BT node to identify the MACs of the devices passing through that section. Additionally, an UIS Gas node and an UIS EP node were deployed at the roundabout center and a Receiver node nearby to receive the frames, processing them and sending the information to the external server (Figure 21). All nodes were installed without the need of any construction work (Figure 22).The SCADA was used to show the information in real time, in other locations.

The tests took place between 10:00 and 14:20, with regular traffic, and including periods of fluctuating traffic density due to peak hours (at the beginning and the end of the period). The following features were checked in these tests:

- The proper separation between the Bluetooth nodes in order to identify the entry and exit node of each vehicle.
- The performance of ultrasound nodes for vehicle counting.
- The reliability of the frame reception of the UIS transmitter nodes at the UIS Receiver node, as well as their coverage in different situations.
- The data obtained from the UIS Gas nodes and UIS EP nodes.
- The performance of the SCADA systems with real data.

In general, the UIS worked properly, counting the vehicles which passed through each section by the UIS ULT nodes, as well as identifying the vehicles’ MAC, although some coverage problems were found with UIS BT node 2 during some moments of the experiment. Environmental and atmospheric pollution parameters have also been monitored, such as O_2_, CO, CO_2_, VOC, noise, light, dust... All this information was shown through the SCADA system.

**Table 4:** *B* matrix obtained through MAC analysis compared with real trips.

	MAC Analysis (*B_MAC_*)				Video Recording (*B_video_*)			
Origin/Destination	1	2	3	4	1	2	3	4
1	10.59	55.29	22.35	11.76	12.61	43.61	21.89	21.89
2	13.36	0.72	31.77	54.15	1.83	2.65	13.03	82.49
3	28.04	63.55	3.74	4.67	22.19	70.10	0	7.70
4	11.11	72.22	16.67	0	6.65	80.59	10.91	1.85

The obtained data served to compute an O-D matrix according to the method described in Section 4. A video recording was made to check the real behavior of vehicles during the experiment. Table 4 shows the *B* matrix obtained by means of the proposed system besides the *B* matrix observed through the video recordings, with results expressed in percentages for an easier interpretation. It can be noticed that differences are limited. The norm for the error matrix (*||B_MAC_-B_video_||_2_*) is 37.72, and the relative error (*||B_MAC_-B_video_||_2_/||B_video_||_2_*) is 0.31.

The B matrix is used to compute the O-D matrix as mentioned above. Table 5 shows the comparison in the same terms as above (writing only one significant digit, due to the relative error, in the computed origin-destination matrix). The norm for the error matrix (*||ODM_MAC_* − *ODM_video_||_2_*) is 703.1, and the relative error (*||ODM_MAC_* − *ODM_video_||_2_/||ODM_video_||_2_*) is 0.42.

**Table 5:** O-D Matrix obtained through UIS compared with real observations.

	MAC analysis (*ODM_MAC_*)				Video recording (*ODM_video_*)				
Origin/Destination	1	2	3	4	1	2	3	4	Trips with origin *i* (*E_i_*)
1	100	300	100	100	72	249	125	125	*571*
2	300	10	600	1.000	39	52	256	1.611	*1.958*
3	200	500	30	40	170	537	0	59	*766*
4	100	800	200	0	72	872	118	20	*1.082*
Trips with destination *j (U_i_)*	*700*	*1.610*	*930*	*1.140*	*353*	*1710*	*499*	*1815*	

These data have been obtained using ultrasound and Bluetooth sensors. In separate field tests with ultrasound sensors, these obtained an accuracy of 98% (see Section 5.2). As for Bluetooth sensors, the separate tests (see Section 5.1) showed that they can identify from 9.88% to 12.48% of the vehicles. This figure takes into account the total number of vehicles, and not the vehicles with a built-in Bluetooth, since it was not possible to obtain a practical way of finding how many of the observed vehicles in a real road have such a device. Besides, no statistics were found about that in the area of the experiments.

A source of error is the low number of trips in some cells (*i.e.*, origin-destination combinations). The number of vehicles moving between a particular combination of origin and destination cannot be controlled in a real experiment. In some cases, no trips were registered (e.g., *ODM_video33_ =* 0). In these combinations, estimations cannot be accurate, and these cells drive a bigger figure for the norm of the relative error, which takes into account all the cells in the matrix. Besides, Bluetooth detection can identify only a proportion of the vehicles, thus reducing the sample size used for the estimations. The magnitude of this reduction depends on the degree of penetration of Bluetooth devices [26], but no figures about penetration of built-in Bluetooth devices in vehicles have been found in the literature. The use of the penetration rate of smartphones could be an alternative, but such a figure should be considered only as a maximum, since the user has to decide to connect the device, and allow it to be visible. However, other studies proposing Bluetooth identification may be valid references. In these cases, the proportion of detected devices varies from 1% to 10% [22,25,27]. The figure obtained in the experiments with the UIS (Section 5.1.2) is consistent with that, identifying from 9.88% to 12.48% of the vehicles, as mentioned above. This proportion is related to the penetration rate of Bluetooth devices, which is considered to increase in the next future, according to Friesen *et al.* [19]. That study reports also a case where a sample of 5% of vehicle identification via Bluetooth has been recognized to provide useful information on traffic flow, as long as data are updated along time.

Another source of deviations in the calculation is related to the loss of coverage in one of the Bluetooth nodes. As mentioned above, a loss of coverage in node 2 was identified during some moments of the experiment. It can be noticed that figures for origin or destination 2 show bigger differences, which may be related to this.

The results show the potential to characterize traffic trends. Despite the moderate time for the experiment, and the low number of vehicles in some origin-destination combinations, the percentage of trips identified by means of Bluetooth allows for a calculation of the O-D matrix. This calculation is performed dynamically, and is available in real time through the SCADA system. Alternative methods like roadside interviews require an extensive amount of time to obtain a comparable sample size, plus the time for processing.

## 6. Conclusions

This work presents a wireless sensor network designed to characterize urban traffic parameters. In particular, vehicle counting and identification are the key to obtain information about origins and destination of trips in the area under study. This data allows for the calculation of the origin-destination matrix in real time, providing the city managers with a powerful tool to adapt traffic planning to real demands. Up to now this matrix required an extensive field work linked to roadside interviews, and needing weeks or months to be completed.

The proposed system has been validated through experiments in real conditions. Transmitter nodes have been tested separately, and data about detection using ultrasound and Bluetooth have been obtained. Experiments with the complete system have also carried out, proving its capability to calculate an origin-destination matrix in real time, with acceptable accuracy. Although the UIS was placed around a roundabout for the experiment, defining the origins and destinations as the different entry and exit ways, it is possible to deploy the system by a wider geographical area, as a city. Besides, calculations can be refreshed in real time through the SCADA system, permitting to traffic managers to obtain an information that up to this moment, required weeks or months.
